# Tumefactive demyelination in older adults: A scoping review of published cases

**DOI:** 10.1177/20552173261416215

**Published:** 2026-04-03

**Authors:** Carla De Pasquale, Todd A. Hardy

**Affiliations:** 3959 Hornsby Ku-Ring-Gai Hospital, NSW, Sydney, Australia;; 560412 Northern Beaches Hospital, Macquarie University, NSW, Sydney, Australia; MS & Neuroimmunology Clinics, Concord Hospital, 2659 University of Sydney, NSW, Sydney, Australia;; Brain & Mind Centre, 90098 University of Sydney, NSW, Sydney, Australia

**Keywords:** Multiple sclerosis, MS, demyelinating, pseudotumor, MRI, diagnosis, ADEM, age, CSF, MOGAD, NMOSD, atypical, late onset

## Abstract

Tumefactive demyelinating lesions (TDLs) are rare in older populations. In this scoping review we conducted a PUBMED search for published cases of TDLs in patients aged ≥55 years. We identified 31 cases and report data on their presentation, diagnosis, treatment, and outcomes. Motor symptoms were the most common presenting symptoms. Investigations including magnetic resonance imaging (MRI) of the brain and spinal cord, CSF studies, serum autoantibodies and brain positron emission tomography (PET) were used to differentiate TDLs from key differentials including malignancy. Key diagnostic findings were similar to those in younger patients with TDLs, with a slightly higher occurrence of open ring enhancement on MRI (11 cases, 35% in present study; 21% in non-age specified populations), and a lower frequency of unmatched oligoclonal bands (6 cases, 19%; 62% in non-age specified populations). The median time to diagnosis was 30 days with 68% (21 cases) of cases requiring biopsy. Initial treatment involved high dose corticosteroids in 90% of patients (29 cases), with 26% (8 cases) started on an MS disease modifying therapy after the index attack. The median Expanded Disability Status Scale score at presentation was 3 and at last follow up was 2.5 indicating potential for limited recovery from TDL in older patients.

## Introduction

Tumefactive demyelinating lesions (TDLs) are large demyelinating lesions in the brain which commonly resemble neoplastic lesions on neuroimaging.^1,2^ Multiple sclerosis (MS) is the most common underlying cause, but TDLs may occur as a clinically isolated syndrome or a radiologically isolated syndrome, as a paraneoplastic phenomenon, or as part of another inflammatory demyelinating disease such as acute disseminated encephalomyelitis (ADEM), neuromyelitis optica spectrum disorder, or myelin oligodendrocyte glycoprotein antibody-associated disease (MOGAD).^2–4^

TDLs can present in individuals with or without previous demyelinating disease and, similar to MS, occur more commonly in females.^1^ The clinical presentation of TDLs can resemble a typical MS relapse. Depending on the location of the lesion in the brain, there may be focal weakness, sensory disturbance or ataxia. A TDL may also present with symptoms and signs atypical for MS-related demyelination, such as headache, seizures, aphasia or confusion – features that reflect the larger size of the lesion, and which are more likely if the lesion is accompanied by oedema and mass effect.^2^

Diagnostic uncertainty about TDLs can lead to significant unease in patients and clinicians and result in unnecessary invasive investigations such as brain biopsy or resection.^2,5^ Ancillary testing including MRI of the spine, CT brain, CSF white cell count and oligoclonal bands, and FDG-PET study may provide clues that suggest a demyelinating lesion over a neoplasm, while serological testing for MOG IgG or aquaporin-4 IgG can help to identify non-MS causes of TDLs.^2^

TDLs occur most commonly in the fourth and fifth decade of life with a mean age of onset of 38.5 +/-15 years.^6^ Therefore, when a TDL presents in an older patient, it is often not even considered as a diagnostic possibility, yet TDLs can occur late in life.^7^ We present 31 cases of TDL in adults aged ≥55 years taken from 27 reports in the literature. The aim of this study is to better understand the demographics, presentation, investigation findings, treatment, and outcomes of patients aged ≥55 with TDLs and to promote awareness of TDLs in older patients.

## Methods

### Study design and ethics

We conducted a retrospective study of published cases identified in PubMed following PRISMA scoping review guidelines including 4 cases recently published by the authors.^7^ The data contained within the studies were already publicly available and anonymised.

### Information sources

**Figure 1.**
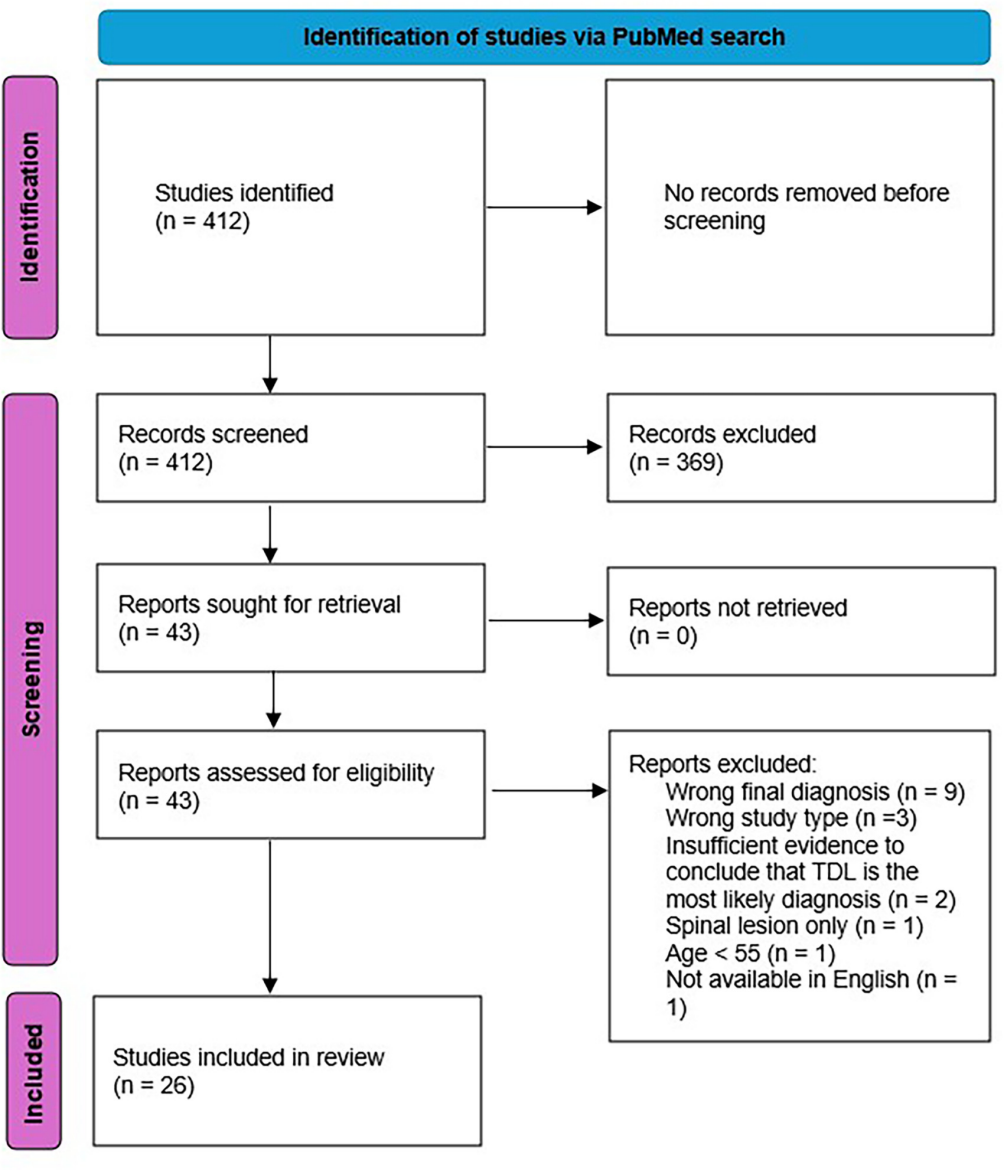
Study selection flowchart.

We searched PubMed with the search terms “tumefactive multiple sclerosis” OR “tumefactive demyelinating lesion” OR “pseudotumoral multiple sclerosis” OR “pseudotumoral demyelinating lesion” OR “tumefactive demyelination” OR “pseudotumoral demyelination” AND “case report” or “case series” OR “case study” from database inception until August 2024, limiting the results to studies available in English. The search results yielded 412 studies. Search results were imported into Covidence (Veritas Health Innovation, Melbourne, Australia), an online software for managing systematic reviews. Each abstract was screened by one reviewer against pre-specified inclusion and exclusion criteria. Forty-three full texts were subsequently screened, and 26 studies identified (Figure 1). One further study known to the reviewers but not yet published was added.

### Inclusion and exclusion criteria

Inclusion criteria

– Case study/case report/case series
– Adult patient aged 55 years or older
– Final diagnosis of tumefactive demyelinating lesion(s) in the brain
– Full text available
– English language study

Exclusion criteria

– Articles in which case details were not presented e.g., narrative review articles
– Patient aged less than 55 years
– Large demyelinating lesion only present in spinal cord
– Final diagnosis of Balo's concentric sclerosis, neuromyelitis optica spectrum disorder, myelin oligodendrocyte glycoprotein antibody-associated disease
– Full text not available (including posters, abstracts)
– Text not available in English
– Insufficient evidence presented in the text to conclude that the TDL is the most likely diagnosis

### Data extraction

The extracted data from these cases for analysis included demographics, clinical presentation, diagnosis, MRI lesion appearance and other investigation results, treatment and outcomes. The initial and final Expanded Disability Status Scale (EDSS) score was estimated by the authors using the information provided in each case.

### Statistical analysis

Continuous data was calculated as mean, median and range. Categorical data was expressed as a proportion of the total number of patients.

## Results

The data from individual cases is recorded in Table 1 (presentation and diagnosis), Table 3 (Investigations), and Table 4 (treatment and outcome).

**Table 1.** Patient demographics and initial diagnosis.

Patient number	First author surname, and year of publication	Age/sex	Medical history	Preceding vaccination	Presenting Symptoms	Initial differential diagnosis	Final diagnosis	Duration from presentation to diagnosis
1	Thebault 2019^23^	62/M	Metastatic seminoma	Not specified	Acutely progressive headache, right-sided weakness, and subtle receptive aphasia	Low grade glioma	Tumefactive demyelination	Not specified
2	Michel 2011^24^	63/F	Sjogren's syndrome	Not specified	Subacute right hemiparesis and Broca's aphasia	CNS demyelinating disease	Pseudotumoral demyelination	<1 week
3	Masu 2009^25^	64/M	Not specified	Not specified	Rapidly progressive hemiparesis	Glioblastoma	Tumefactive demyelinating plaque	Not specified
4	Ueno 2020^26^	60/M	Hypertension, lumbar stenosis, and chronic inflammatory demyelinating polyneuropathy with onset six months prior, receiving monthly IVIg for past 3 months	Not specified	Visual impairment in left eye and sensory disturbance on left side of face	Not specified	Combined central and peripheral demyelination	<1 week
5	Heyman 2001^27^	61/F	Treated hypertension	Not specified	Amblyopia	Retrobulbar neuritis	Inflammatory demyelination	>1 month
6	Hane 2011^28^	70/M	Coronary heart disease, atrial fibrillation, renal cell carcinoma	Not specified	Confusion, headache, hemineglect	Neoplasm	Recurrent tumefactive demyelination	Not specified
7	Rogers 2018^29^	56/F	Hypertension, Hashimoto thyroiditis	Not specified	Left-sided facial numbness, word-finding difficulty, and ataxia	Acute demyelination or metastatic disease	Acute demyelination	>1 month
8	Hashimoto 2019^30^	60/M	Not specified	Not specified	One month left lower limb sensory loss; right visual field	High grade astrocytoma	Tumefactive MS	>1 month
9	Hashimoto 2019^30^	74/F	Rheumatoid arthritis on adalimumab	Not specified	Sudden left facial paralysis and left hemiplegia 4 days later	Not specified	Tumefactive MS	Not specified
10	Nakamura 2017^31^	57/F	None	Not specified	One month slowly progressive left-sided hemiparesis	Brain tumour	Tumefactive demyelination	>1 month
11	Broadfoot 2015^32^	60/M	Retroperitoneal germ cell cancer	Not specified	Four weeks of visuospatial problems and memory loss	Glioma or lymphoma	Paraneoplastic tumefactive demyelination	1 week – 1 month
12	Siffrin 2014^33^	55/M	Paroxysmal atrial fibrillation	Not specified	Right-sided ataxia predominantly of the right leg, positive pyramidal signs on the right and subacute mild aphasia	Glioma	Tumefactive demyelinating disease	Not specified
13	Krouwer 1998^34^	59/M	Previous demyelinating disease 7 months earlier	Not specified	Blurred vision, unsteady gait, right homonymous hemianopsia, nystagmus, right facial palsy, dysarthria, and left dysmetria	Neoplasm	Demyelinating disease	Not specified
14	Sacco 2021^35^	67/F	Three days of upper respiratory tract infection, fever, and headache	Not specified	Acute onset of speech impairment and drowsiness	Left middle cerebral artery stroke	Fulminant inflammatory demyelination	<1 week
15	Selkirk 2005^36^	60/M	Multiple sclerosis, sleep apnoea, depression	Not specified	Balance and coordination problems, unsteady gait, progressive behavioural changes, apathy, social withdrawal, urinary incontinence and fatigue	MS	Tumefactive MS exacerbation	<1 week
16	Takeuchi 2008^37^	87/F	Uterine cancer with hysterectomy, lymphadenectomy and external beam radiation 35 years prior; total hip replacement due to femoral neck fracture 6 years prior; ruptured bladder with surgical repair	Not specified	Two weeks of worsening hemiparesis, dysarthria	MS, malignant lymphoma, metastatic tumour	Tumefactive MS	Not specified
17	Weil 2022^38^	72/F	Obesity, hypertension, hyperlipidaemia, non-alcoholic steatohepatitis, erythema nodosum	Second dose of mRNA COVID-19 vaccine (Moderna) one week prior	Five weeks of progressive left-sided weakness and numbness, mild dysarthria, left facial weakness and moderate left-sided hemiparesis with hemisensory loss	Tumefactive demyelinating lesion	Recurrent tumefactive demyelinating lesion	>1 month
18	Boyle 2023^39^	55/M	Recent oral HSV	Influenza vaccination 3 weeks prior	Groin pain, acute dysarthria, apraxia, and left hemiparesis	Not specified	Unspecified tumefactive demyelination	Not specified
19	Golombievski 2015^40^	69/M	Hypertension, dyslipidaemia, coarctation of aorta (diagnosed at age 18 and corrected with surgery), and L4-L5 laminectomies	Not specified	Two-months of dysgraphia, poor coordination of his right hand, and word finding difficulties	Not specified	Tumefactive demyelinating lesion	Not specified
20	Garg 2022^41^	56/M	None (concomitant glioma identified during index attack)	First dose of adenovector based ChAdOx1 (COVISHIELD_TM_) vaccine 2 days prior	Right hemiparesis, hypertension	Not specified	Tumefactive demyelinating lesion	Not specified
21	deMedeiros 2014^42^	55/F	Ovarian cancer	No	Fatigue and discrete bilateral retroorbital pain, followed by homonymous hemianopia and tetrahyperreflexia	Primary tumour (glioblastoma, lymphoma), metastasis from ovarian cancer, or infectious, inflammatory, or pseudotumoral demyelinating process	Pseudotumoral multiple sclerosis	<1 week
22	Nicholas 2016^43^	57/M	Not specified	Not specified	Left sided facial droop and expressive aphasia	Stroke; thyroid cancer with brain metastasis	MS	Not specified
253	Maia 2021^44^	63/F	T2DM, dyslipidaemia	No	Four days evolution of lower limb weakness, constipation and urinary retention	Inflammatory demyelinating process	Marburg variant multiple sclerosis	>1 month
24	Shishido-Hara 2021^45^	69/F	Not specified	Not specified	Disorientation while driving	Demyelinating disease	Tumefactive demyelinating disease	<1 week
25	Villarreal 2021^46^	62/M	Not specified	No	Aphasia, acute cognitive changes	Not specified	Tumefactive MS	Not specified
26	Conforti 2016^47^	66/F	Non-communicating syringomyelia	Not specified	Fifteen days left occipital pain radiating to frontal lesion with acute sudden onset paraphasia	Suspicious for tumour	Late-onset tumefactive MS with an isolated TDL	>1 month
27	Tosunoglu 2024^48^	55/F	None	Not specified	Right-sided headache, blurred vision, occasional numbness, and loss of strength in her right arm and leg with onset 2 years prior	Tumefactive MS	Tumefactive MS	<1 week
38	De Pasquale 2025^7^	67/F	Gastro-oesophageal reflux	Influenza vaccination one month prior, Covid-19 vaccination 2 months prior	One month of cognitive decline, headache, left-hand weakness and numbness, left facial droop, urinary urgency, and falls	Malignancy	Tumefactive demyelinating lesion	>1 month
29	De Pasquale 2025^7^	72/M	Hypertension, hyperlipidaemia, and mitral valve prolapse	Zostavax and FluQuadri vaccinations 3 months prior	One day of right-sided visual loss and expressive dysphagia	Lymphoma, glioma	Tumefactive demyelinating lesion	>1 month
30	De Pasquale 2025^7^	82/M	Emphysema, rheumatoid arthritis	No	Dysarthria	Stroke, malignancy	MS with a TDL	>1 month
31	De Pasquale 2025^7^	70/M	Idiopathic neutropaenia, hyperlipidaemia, and Meniere's disease	No	Subacute onset left hemiparesis with pseudoathetoid movements of the left hand and left-sided neglect	Subacute ischaemic stroke, neoplastic lesion, CNS lymphoma, atypical MS, seizure with Todd's paralysis	Tumefactive demyelinating lesion	1 week- 1 month

CNS: central nervous system; CSF: cerebrospinal fluid; EDSS: expanded disability status scale; IV: intravenous; IVIg: intravenous immunoglobulin; MRI: magnetic resonance imaging; mRNA: messenger ribonucleic acid; MS: multiple sclerosis; PET: positron emission tomography; T2DM: type 2 diabetes mellitus; TD: tumefactive demyelination; TDL: tumefactive demyelinating lesion; WCC: white cell count

### Patient demographics

Thirty-one cases were identified from a total of 27 reports. The median age at presentation was 62 years (range 55–87 years). Fifteen patients (48%) were female. Two patients (6%) had a pre-existing diagnosis of MS. Five cases (16%) noted vaccination in the preceding three months. Four cases (13%) had underlying or concomitant active malignancy – seminoma, renal cell carcinoma, retroperitoneal germ cell tumour, and glioma (Table 1, cases 1, 6, 11, and 20 respectively). The patient demographics are summarised in Table 2.

**Table 2.** Patient demographics.

Median age in years (standard deviation, range)	62 (1.4, 55–87)
Female sex	15 (48%)
Pre-existing MS	2 (6%)
Vaccination in previous 3 months	5 (16%)
Underlying/concomitant malignancy (seminoma, renal cell carcinoma, retroperitoneal germ cell tumour, and glioma)	4 (13%)

### Presentation

**Figure 2.**
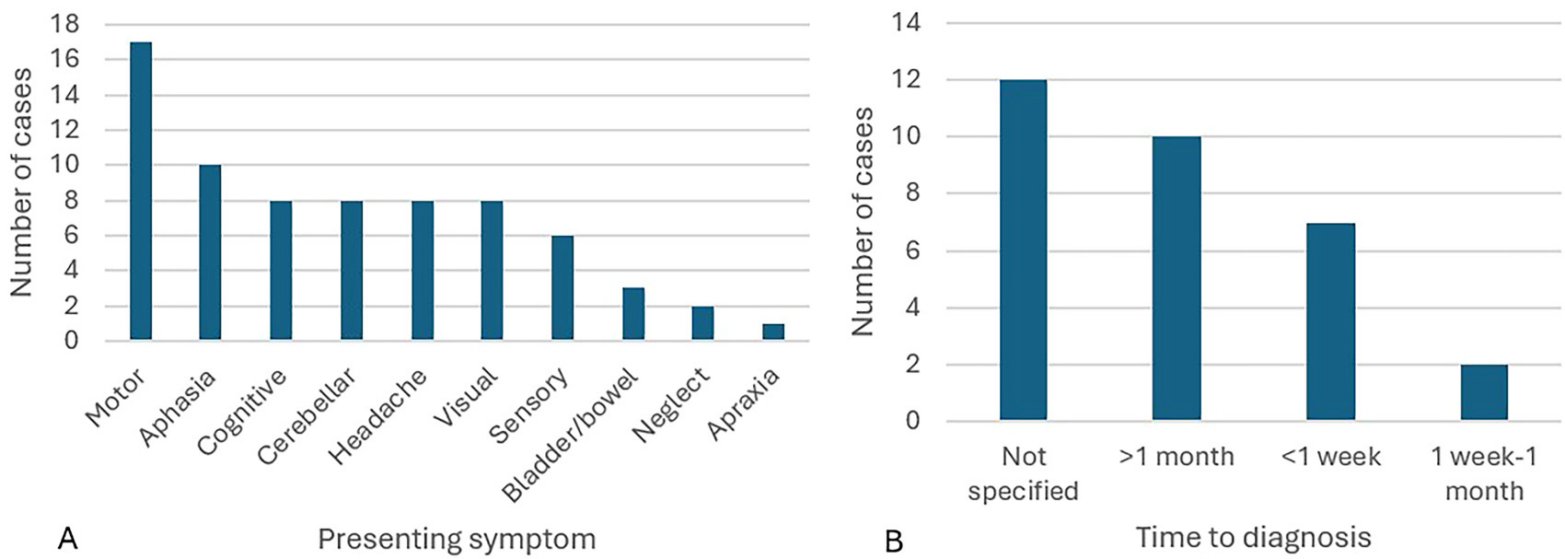
(A) Presenting symptoms; (B) time from presentation to diagnosis.

The most common presenting signs and symptoms were motor (17 cases, 55%), aphasia (10, 32%), cognitive (8, 26%) and cerebellar (8, 26%) (Table 1; Figure 2.A). In 16 cases (52%), the initial differential diagnosis included malignancy, and in 10 cases (32%) the initial diagnosis included demyelination. In the 19 studies that specified the time from presentation to diagnosis the median time to diagnosis was 30 days (SD = 24.5 days), with 10 (32%) of cases requiring >1 month to arrive at a diagnosis (Figure 2.B).

### MRI findings

**Figure 3.**
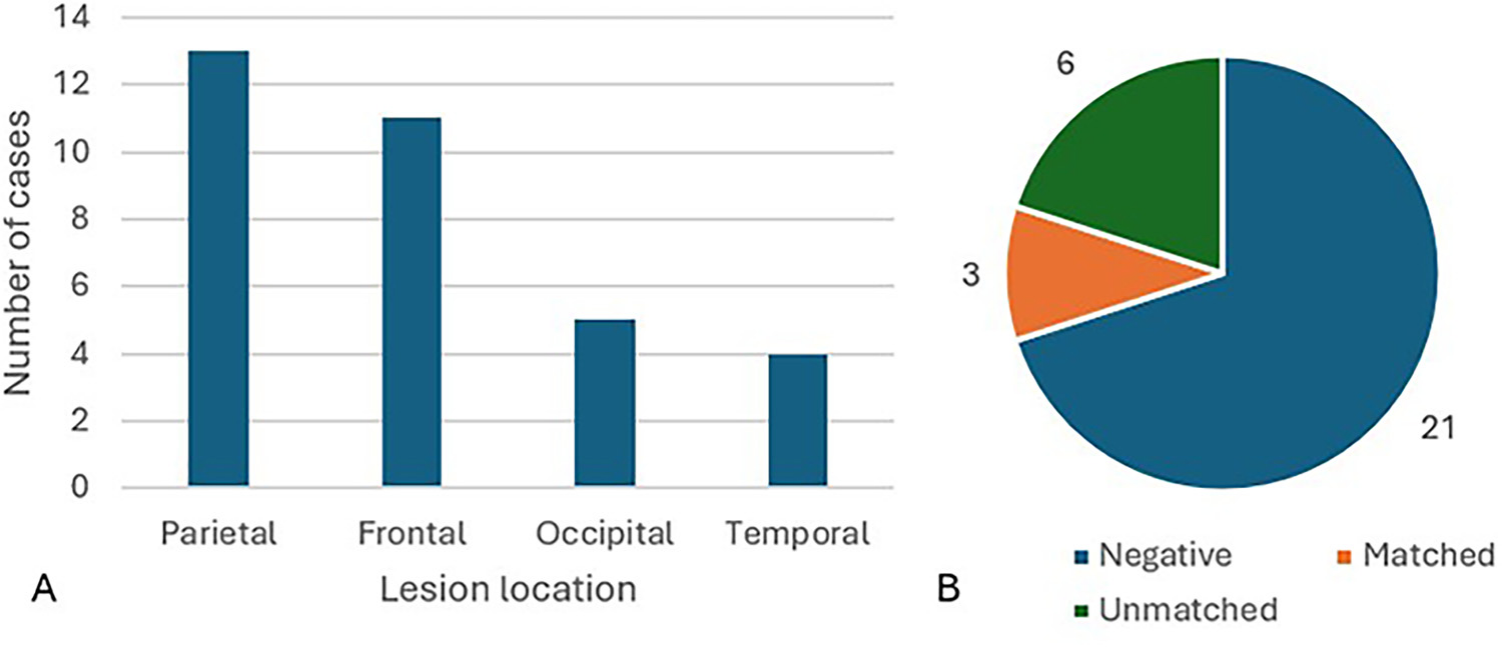
(A) Lesion location on MRI; (B) oligoclonal bands of IgG in CSF analysis.

The most affected brain areas seen on MRI were the parietal (13 lesions, 42%) and frontal lobes (12 lesions, 35%) (Figure 3.A). Enhancement with intravenous gadolinium contrast was seen in 28 lesions (90%) with 13 of these (46%) noting ring enhancement, 11 of which specified open or incomplete ring enhancement (39%). The median lesion size in the 15 cases where dimensions were reported was 33mm (SD = 4.7, range 14–70). Other lesions were present in 10 cases (32%). MRI spine at presentation was reported in 9 cases (29%), 4 of which identified a spinal lesion (13% of all patients).

### CSF findings

CSF results were included in 30 cases (97%), with 21 of these negative for OCBs (68%), 6 were positive and unmatched in the serum and CSF (19%) and 3 were positive and matched (10%) (Figure 3.B). Protein was elevated in 17 of the 26 studies in which it was reported (65%). In the 18 studies which reported the CSF protein value, the median was elevated at 695 mg/L (SD = 89.8 mg/L, range 210–1960 mg/L). WCC was elevated in 11 of the 23 (46%) studies in which it was reported. (Using New South Wales Health reference range 150–450 for protein, <1 for WCC). Two studies specified CSF lymphocyte predominance (Table 3, cases 6 and 15) and one specified neutrophil predominance (Table 3, case 18). Where a value was reported, the median WCC was 0 /mm^3^ (SD 32.5, range 0–718 /mm^3^); the median WCC among studies in which WCs were elevated at 1/mm^3^ or more was 1.5 /mm^3^ (SD = 51.0, range 0–718).

**Table 3.** Investigations.

Patient number	MRI findings (Location, size, enhancement pattern)	Other lesions present	MRI spine	CSF findings (oligoclonal bands, protein (mg/L), WCC (x 10^6/L))	Brain PET	Biopsy findings
1	Left subcortical frontoparietal, minimal focal enhancement	No	Not specified	Negative, normal, normal	Not completed	Demyelination and inflammatory infiltration with a predominance of CD68-immunoreactive foamy reactive macrophages
2	Left corona radiata, incomplete ring enhancement	Yes	Not specified	Unmatched, 710, not reported	Not completed	Not completed
3	Right basal ganglia, 37mm, peripheral enhancement	No	Not specified	Negative, normal, normal	Not completed	Typical histological features of an acute demyelinating plaque
4	Left pons/middle cerebellar peduncle, open ring enhancement	Yes	Not specified	Negative, 980, normal	Not completed	Not completed
5	Right subcortical Rolandic, incomplete enhancement with well-defined margins	No	Not specified	Negative, normal, normal	Not completed	Inflammatory demyelination
6	Right parieto-occipital	Not specified	Not specified	Negative, 710, 27 (lymphocyte predominant)	Not completed	Foamy macrophages and focal perivascular lymphocytic cuffs, indicative of demyelination
7	Right cerebellum, enhancement	Yes	Not specified	Negative, not tested, not reported	Not completed	Demyelination
8	Pons, no enhancement	Yes	Not specified	Negative, elevated, elevated	Hypometabolic	Loss of myelin, relative preservation of axons and infiltration of foamy macrophages without tumour cells
9	Right frontotemporal, 70×50mm, ring enhancement	No	Not specified	Negative, 320, 4	Hypermetabolic	Loss of myelin and relative preservation of axons. Infiltration of foamy macrophages without tumour cells
10	Right frontal, open ring enhancement	No	Normal	Negative, 620, not reported	Not completed	Loss of myelinated fibres with relative axonal preservation, lipid-laden macrophages, bizarre astrocytes, and perivascular lymphocytic inflammatory infiltration, occasional Creutzfeldt cells, consistent with tumefactive demyelination
11	Bilateral occipitoparietal, patchy enhancement	No	Not specified	Negative, normal, normal	Not completed	Active demyelination with hypercellular white matter, a dense infiltrate of CD68-positive foamy macrophages, some containing myelin debris, and scattered CD45+ and CD3+ lymphocytes but no evidence of neoplasia
12	Left thalamus, marked but irregular enhancement	No	Not specified	Negative, not reported, 8	Not completed	Lymphocyte infiltration with predominant T cell and less B cell composition. Almost complete demyelination and relative preservation of axons, suggestive of inflammatory demyelinating disease reminiscent of classic multiple sclerosis
13	Left parietooccipital, enhancement	No	Not specified	Unmatched, moderately elevated, normal	Not completed	Demyelination, reactive gliosis, infiltrates of foamy lipid laden macrophages and mononuclear cells
14	Left frontal involving corpus callosum, peripheral open-ring enhancement	No	Normal	Matched, elevated, 11 (lymphocyte predominant)	Not completed	Not completed
15	Right periventricular, 40×35mm, ring enhancement	Yes	T2 hyperintensity at L4 / L5 levels	Unmatched, 740, not reported	Hypometabolic	Not completed
16	Left temporoparietal, open ring enhancement	Yes	Not specified	Negative, 680, not reported	Not completed	Proliferation of foamy macrophages and reactive astrocytes with abundant cytoplasm and eccentric nuclei, numerous positive macrophages in the white matter, and absence of the myelin sheath
17	Right frontoparietal, 47mm, partial ring enhancement	No	Not specified	Negative, normal, normal	Not completed	Areas of reactive gliosis with dense infiltration of foamy, CD68 positive macrophages predominantly involving the white matter with relative preservation of axons; consistent with a non-neoplastic, demyelinating process
18	Right posterior frontal, 55, peripheral enhancement	No	Not specified	Negative, 1010, elevated (neutrophil predominant)	Not completed	Not completed
19	Left frontal, 20×23 mm, ring enhancement	Not specified	Not specified	Unmatched, 770, 0	Not completed	Reactive gliosis with CD68 macrophages most likely consistent with a demyelinating lesion
20	Left parietal	No	Not specified	Not reported	Not completed	Not completed
22	Left temporo-occipital, 37×24×18 mm, open ring enhancement	No	Spinal lesion identified later at T2-3 level	Negative, 460, 1	Not completed	Well-demarcated areas of demyelination in the white matter, a reduction in the number of oligodendrocytes, relative axonal preservation, diffuse infiltration by foamy macrophages, mild perivascular lymphocytic inflammatory infiltration, and the proliferation of reactive gemistocytic astrocytes; consistent with active demyelinating disease
22	Left parietal, 25mm, open ring enhancement	Yes	Not specified	Unmatched, not reported, not reported	Normal	Not completed
23	Multiple lesions in cerebral hemispheres and cord, largest lesion 27mm	Yes	Intramedullary lesion	Negative, 1960, 2	Normal	Perivascular inflammation with massive macrophage infiltration and reactive astrocytes associated with widespread demyelination, severe axonal injury and destruction, consistent with an aggressive form of MS, classically described as Marburg variant
24	Right parietal, arcuate contrast enhancement	No	Not specified	Negative, normal, normal	Not completed	Huge meandering blood vessels at the periphery of the mass-forming lesion, lymphocyte predominant inflammatory cells, angiodestructive inflammation. Phagocytosis of myelin particles was suspected.
25	Right parietal, peripheral enhancement with linear focus	No	Not performed	Negative, 970, 6	Not completed	Perivascular mononuclear infiltrates and reactive gliosis
26	Left frontal, 65×57 mm, mild peripheral enhancement	No	Normal	Negative, 210, 1	Not completed	Hypercellular white matter lesion with predominant macrophages (CD 68+) mixed with hypertrophic astrocytes (GFAP+) presenting moderate atypic nuclei and rare lymphocytes (LCA+ and CD3+). The second histological opinion of the brain specimen described an increased cell number, numerous macrophages infiltrating the lesion, loss of myelin, and reactive astrocytes with swollen cell bodies.
27	Right frontal, 20mm, annular intermediate enhancement	Yes	Short segment lesion at C4 level	Unmatched, not reported, not reported	Not completed	Not completed
28	Right posterior frontal, 33×22 mm, open ring enhancement	No	Normal	Matched, 510, 0	Hypermetabolic lesion with hypometabolic centre	A primary demyelinating process with numerous foamy macrophages, perivascular infiltrates and reactive astrocytosis with no features to suggest ADEM
29	Left temporal, 32×29×22 mm, central area of enhancement	No	Normal	Negative, 839, 0	Hypometabolic	Sheets of macrophages and perivascular lymphocytes, accompanied by myelin loss and relative preservation of axons with rare Creutzfeld astrocytes. There were no perivenous cuffs of T lymphocytes and macrophages associated with oedema to indicate ADEM
30	Left fronto-parietal, 14×12 mm, homogenous enhancement	Yes	Single lesion at C1	Matched, 520, 0	Normal	Scattered prominent gemistocytes and marked infiltrate of foamy histiocytes and T lymphocyte predominant perivascular and mild interstitial lymphocytic inflammation. A Creutzfeldt cell was noted
31	Right parieto-occipital, 15×12 mm, mild and poorly defined peripheral enhancement	No	Normal	Negative, 390, 1	Hypometabolic	Primary demyelination with numerous macrophages, some reactive astrocytes, and scanty perivascular and interstitial lymphocytes

Key: ADEM: acute disseminated encephalomyelitis; CD: cluster of differentiation; CNS: central nervous system; CSF: cerebrospinal fluid; EDSS: expanded disability status scale; GFAP: glial fibrillary acidic protein; IV: intravenous; IVIg: intravenous immunoglobulin; MRI: magnetic resonance imaging; MS: multiple sclerosis; PET: positron emission tomography; TD: tumefactive demyelination; TDL: tumefactive demyelinating lesion; WCC: white cell count

### Other testing

Investigation for serum MOG IgG and aquaporin 4 IgG were tested in 11 and 15 cases respectively (35%, 43%), all of which were negative. Brain PET was included in 9 cases (29%), 4 of which showed a hypometabolic lesion (44%), 2 showed a hypermetabolic lesion (22%), and 3 were normal (33%). The lesion was biopsied in 21 cases (68%).

### Initial treatment

Corticosteroids were the most common initial treatment, utilised in 28 cases (90%), with 18 cases (58%) specifying the use of high dose intravenous corticosteroids. The lesion was resected in 4 cases (13%).

### Longer term treatment and follow up

The median length of available follow up was 12.5 months. Eight patients (26%) experienced further demyelinating lesions at last follow up., 5 of these were symptomatic (63%), 2 were identified on follow up MRI (25%), and 1 was not specified (13%). The median duration of follow up in patients who had further lesions was 16 months (SD = 2.6 months, range 0–48 months). Twelve patients (39%) received long-term immunosuppression of some description (Table 4); eight of which were started after the first episode (25%), three after a second episode (10%), and one was pre-existing (3%) for another indication (rheumatoid arthritis) with 8 of these (26%) started on disease modifying MS therapy (DMT). The median initial EDSS was 3.0 (SD = 0.34, range 0–6.5); nineteen patients (61%) demonstrated an improved EDSS at last available follow up; six patients (19%) demonstrated a worsened EDSS, and three (10%) demonstrated no change. Where estimates were available, the median change in EDSS from presentation to last follow up was an improvement to an EDSS of 1.0 (SD = 0.48, range 0–11.5).

## Discussion

We present data collected from 31 published cases of TDLs in patients aged ≥55 years. In our study, we saw slightly more male than female cases which differs from the slight female predominance seen in non-age specified tumefactive demyelination, but is in keeping with studies that have identified a less striking female predominance of older patients presenting with late onset MS (LOMS) compared to early onset MS.^8^ There appeared to be a lower index of suspicion for TDLs in older patients, with only 32% of the initial differential diagnoses including demyelination, compared to 70% among patients of all ages.^6^ Again, this is mirrored in cases of LOMS, where similar delays in diagnosis have been recognised.^9^ Likely as a result, the frequency of patients undergoing brain biopsy was 68% among our cases which is significantly higher than the non-age specified rate of 30%.

There were minor differences in the investigation results in our cohort of older patients compared to 257 non-age specified TDL cases^6^ (Table 5). Cerebral MRI findings were similar across both groups, with frontal and parietal lesions comprising the largest proportion of cases. Incomplete open ring enhancement; one of the most specific radiological signs for TDLs, was present in 35% of cases – higher than that of non-age specified cases (21%).^10^ CSF-restricted oligoclonal bands were positive less frequently in the aged cohort (19% compared to 62% in a mixed aged cohort). CSF protein was more commonly elevated in older patients, although it is worth noting that CSF protein increases with age anyway.^11^ We note that these findings have not been demonstrated in comparisons between LOMS and early onset MS (EOMS) patients, though this may be partially attributable to the exclusion of clinically isolated syndromes.^9^ Brain PET was infrequently utilised. When used, 78.4% of cases showed a normal or hypometabolic lesion which is more typical of a TDL than a high-grade CNS malignancy.^12^

**Table 4.** Treatment and outcome.

Patient number	Resection	Initial treatment	Long-term immunosuppressive therapy	Duration of follow up (months)	Further demyelinating lesions	Estimated EDSS initial/at last follow up
1	Yes	High dose dexamethasone	No	10	Yes	3/4
2	No	Nine months IV and oral steroids	Hydroxychloroquine	9	No	4.5/improved
3	No	Steroid pulse therapy	No	2	No	4.5/4
4	No	Steroid pulse therapy stepped down to oral prednisolone; plasma exchange	No	1	No	6/3
5	No	IV corticosteroids	No	40	No	2/3
6	No	Oral dexamethasone	No	34	Yes	3/death due to renal cell carcinoma
7	No	Nil	Fingolimod	16	Yes	2/2
8	No	Steroid treatment - unspecified	No	0	No	2.5/not specified
9	No	Corticosteroids	No – although patient was previously on adalimumab for rheumatoid arthritis. No further information is available regarding long-term continuation, cessation, or alternative therapies commenced	0	No	3.5/improved
10	No	3 days IV methylprednisolone 1g daily	Interferon beta after episode of recurrence	16	Yes	3/4
11	No	Dexamethasone 4mg twice daily, then 3 days IV methylprednisolone 1g daily; plasma exchange	No	9	No	4.5/3.5
12	No	Dexamethasone 24mg daily for 10 days, then 5 days IV methylprednisolone 1g daily	Azathioprine, mycophenolate mofetil, cyclophosphamide, rituximab respectively for episodes of recurrence; stabilised with rituximab long-term	26	No	4/3.5
13	No	Corticosteroids, IVIg, plasma exchange, cladribine, cyclophosphamide	Cyclophosphamide	36	No	6/8.5
14	No	Empiric therapy with ceftriaxone and acyclovir, followed by 10 days IV methylprednisolone 1g daily; 2 doses cyclophosphamide	No	24	No	3/1
15	No	Steroid treatment – unspecified	No	5	No	6/4.5
16	No	10 days steroid treatment – unspecified	No	2	No	4.5/3
17	No	IV methylprednisolone for 7 days, then oral prednisolone tapering; 5 sessions plasmapheresis	Rituximab after episode of recurrence	5.5	Yes	6/10
18	No	Empiric dexamethasone 4 mg twice daily, levetiracetam 500 mg twice daily, broad spectrum antibiotics and acyclovir; then 3 days IVIg and 5 days IV methylprednisolone 1g daily; rituximab	No	42	No	8.5/1
19	Yes	5 days IV methylprednisolone 1g daily	No	13	No	2/6
20	No	2 weeks oral methylprednisolone (32 mg/day), then tapering oral dose	No	3	No	5.5/1
21	No	5 days IV methylprednisolone 1g daily	No	36	Yes	2.5/0
22	No	2mg dexamethasone twice daily	No	17	No	3/2
23	No	IV methylprednisolone 1g daily	Natalizumab	12	No	2.5/2
24	No	Steroid treatment – unspecified	No	9	No	2/0
25	No	Not specified	Interferon beta-1b	0	Yes	Not specified
26	No	Dexamethasone and IV mannitol, with tapering oral dexamethasone dose	Cyclophosphamide	12	Yes	2/2
27	No	10 days IV methylprednisolone 1g daily	Interferon beta-1a	0	No	3/1
28	Yes	Dexamethasone	No	15	No	2.5/1
29	No	3 days IV methylprednisolone 1g daily	No	48	No	3/2
30	Yes	3 days IV methylprednisolone 1g daily	Teriflunomide	9	No	2/2
31	No	3 days IV methylprednisolone 1g daily	No	14	No	8.5/6

CNS: central nervous system; CSF: cerebrospinal fluid; EDSS: expanded disability status scale: IV: intravenous; IVIg: intravenous immunoglobulin; MRI: magnetic resonance imaging; MS: multiple sclerosis; PET: positron emission tomography; TD: tumefactive demyelination; TDL: tumefactive demyelinating lesion; WCC: white cell count

We suggest that the use of ancillary testing may provide opportunities to avoid invasive brain biopsy and the associated risks in older patients.^13^ For example, in a patient with a solitary cerebral lesion suspicious for a TDL, and no prior history of MS, MRI of the spine to screen for demyelinating lesions, CSF examination for intrathecal oligoclonal bands, and FDG-PET imaging to help exclude high grade malignancy can provide clues as to whether a lesion is more likely to be a TDL or a malignancy. MR perfusion showing reduced cerebral blood volume within the lesion also favours a TDL over high grade neoplasm and should be considered in cases of diagnostic uncertainty.^14^ Serum autoantibody screening, such as MOG IgG and aquaporin-4 IgG, can be used to diagnose MOGAD or NMOSD respectively.^2^ Less than 50% of the presented cases included results for MOGAD or NMSOD testing respectively, which may also reflect that several of these reports predate the use of these screening assays. This represents a potential source of bias in these results.

**Table 5.** Comparison of results in present study and a non-aged-specified cohort of TD cases.

	Present study (n=31)	Non-age specific TD cases (Fereidan-Esfahani, 2023) (n=257)
**Presentation**		
Median age	62 years	38.5 years
Female	50%	57%
Presenting symptoms	Motor 55% Aphasia 32% Cognitive 26% Cerebellar 26% Headache 26% Visual 26%	Motor 46% Sensory 37% Cerebellar 17%
Demyelination included in initial diagnosis	32%	70%
**MRI findings**		
Median size on MRI	33mm	20.3mm
Most common focus of lesion	Parietal 42% Frontal 35% Occipital 16%	Frontal 42% Parietal 31% Temporal 9%
Incomplete open-ring enhancement	35%	21%
**CSF findings**		
Elevated protein	55%	46%
Elevated WCC	35%	34%
Unmatched OCBs	19%	62%
**Biopsy**	68%	30%
**Treatment**		
Initial treatment with corticosteroid	90%	82%
Started on disease modifying therapy after index attack	26%	48%
**EDSS**		
Median EDSS initial	3.0	4.0
Median EDSS at last follow up	2.5	2.5

CSF: cerebrospinal fluid; EDSS: expanded disability status scale score; MRI: magnetic resonance imaging; OCBs: oligoclonal bands; TD: tumefactive demyelination; WCC: white cell count

The most common initial treatment in our study was corticosteroids which were administered in 90% of cases. This is in keeping with recommendations of three-days of intravenous methylprednisolone followed by repeat imaging in 4–6 weeks to assess response in those cases where a TDL is difficult to exclude from a glioma.^2,^^15,16^ This approach is better avoided where the differential diagnosis lies between TDL and CNS lymphoma as corticosteroids before a diagnosis of CNS lymphoma is established can alter histopathology and lead to false reassurance that a biopsied lesion is not due to lymphoma. We also recommend close clinical and MRI follow-up of biopsy proven cases of TDL as cases of CNS lymphoma arising from sentinel demyelination have been described.^17^ Cases of recurrent or relapsing TDLs should invoke a widespread search for an underlying malignancy.^18^ In patients with a pre-existing diagnosis of malignancy, with the potential for metastasis to the brain, we would advocate a lower threshold for proceeding to biopsy.

The low rate of prescribing of DMTs might reflect that older patients with MS are less likely to have inflammatory disease activity requiring immune intervention,^19,20^ but also less frequently meet the diagnostic criteria for MS. Additionally, older patients may be more vulnerable to the adverse effects of these therapies and this may influence clinical decision making.^21^ Almost two-thirds of cases had improvements in EDSS following their clinical nadir which indicates that older patients can make some degree of recovery from TDLs despite more limited brain reserve. We report radiological relapse in 26% of patients, as compared to 50% in a non-age specified cohort.^22^ Relapse has been identified as more likely in patients with unmatched OCBs, which may be of significance given the low rates of both unmatched OCBs and relapse in the presented cases.^22^ We also note that all of the eight cases with a further MS lesion at last follow up had negative OCBs at their index attack which suggests that OCBs are not a predictor of future relapse in this cohort.

There are several limitations to this study. Only a small number of published cases could be included. Additionally, case reports often contained incomplete datasets and details such as recent vaccination or detailed serological screening were missing. Data points such as time to diagnosis and EDSS were estimated based on the information provided. Whilst this discussion has compared our findings to that of a non-age specified cohort, this comparison is qualitative only and no statistical analysis has been completed.

This review highlights TDLs as a differential diagnosis in older people with a cerebral lesion, noting a broadly similar presentation and investigation findings to that of TDLs in younger people except for a lower frequency of intrathecal OCB synthesis and a higher proportion of open ring enhancing lesions. We suggest that appropriate use of neuroimaging and ancillary testing may help to avoid misdiagnosis and unnecessary biopsy in some patients.
